# Length of pressure-controlled reperfusion is critical for reducing ischaemia-reperfusion injury in an isolated rabbit lung model

**DOI:** 10.1186/1749-8090-2-54

**Published:** 2007-12-07

**Authors:** Stefan Guth, Diethard Prüfer, Thorsten Kramm, Eckhard Mayer

**Affiliations:** 1 Department of Cardiothoracic and Vascular Surgery, Johannes Gutenberg – University, Langenbeckstr. 1, 55131 Mainz, Germany; 2 Diakonie Hospital Rotenburg, Teaching Hospital of the University of Göttingen Elise-Averdieck. Str. 17, 27356 Rotenburg, Germany

## Abstract

**Background:**

Ischaemia-reperfusion injury is still a major problem after lung transplantation. Several reports describe the benefits of controlled graft reperfusion. In this study the role of length of the initial pressure-controlled reperfusion (PCR) was evaluated in a model of isolated, buffer-perfused rabbit lungs.

**Methods:**

Heart-lung blocks of 25 New Zealand white rabbits were used. After measurement of baseline values (haemodynamics and gas exchange) the lungs were exposed to 120 minutes of hypoxic warm ischaemia followed by repeated measurements during reperfusion. Group A was immediately reperfused using a flow of 100 ml/min whereas groups B, C and D were initially reperfused with a maximum pressure of 5 mmHg for 5, 15 or 30 minutes, respectively. The control group had no period of ischaemia or PCR.

**Results:**

Uncontrolled reperfusion (group A) caused a significant pulmonary injury with increased pulmonary artery pressures (PAP) and pulmonary vascular resistance and a decrease in oxygen partial pressure (PO_2_), tidal volume and in lung compliance. All groups with PCR had a significantly higher PO_2 _for 5 to 90 min after start of reperfusion. At 120 min there was also a significant difference between group B (264 ± 91 mmHg) compared to groups C and D (436 ± 87 mmHg; 562 ± 20 mmHg, p < 0.01). All PCR groups showed a significant decrease of PAP compared to group A.

**Conclusion:**

Uncontrolled reperfusion results in a severe lung injury with rapid oedema formation. PCR preserves pulmonary haemodynamics and gas exchange after ischaemia and might allows for recovery of the impaired endothelial function. 30 minutes of PCR provide superior results compared to 5 or 15 minutes of PCR.

## Background

Since the middle 1980s lung transplantation (LTX) has become an accepted treatment option for patients with end-stage pulmonary disease, but ischaemia reperfusion (IR) injury of the pulmonary graft is still a serious early problem after LTX [1].

Ten to 20% of transplanted lung allografts develop a severe graft dysfunction (IR-injury) that yields to a high early mortality and ongoing morbidity [2-6]. The clinical features are pulmonary oedema with diffuse infiltrates in the chest radiographs, decreased lung compliance (C_L_), progressive hypoxaemia, and an increased pulmonary vascular resistance (PVR) [7,8]. Histological examinations show alveolar damage, interstitial oedema and sequestration of neutrophils [9]. Waddell and others reported that IR-injury predisposes grafts to early rejection by up-regulation of class II major histocompatibility antigens [10], chronic bronchiolitis obliterans syndrome resulting in graft failure [11].

In the past multiple methods of graft preservation [8,12-14] have been evaluated. However, the major injury may occur within the first minutes of reperfusion [15-17]. Interventions in the early reperfusion period, for example inhalation of NO [1], suppression of neutrophils [9,18], decrease of oxygen radical generation [18], and pressure-controlled reperfusion (PCR) [19] showed favourable effects on IR-injury. Despite the documented effectiveness of this approach after myocardial ischaemia [20] or ischaemia of the lower extremities [20,21] equivalent data about controlled reperfusion of the lungs are rare. In other reports beneficial effects of controlled reperfusion by changing the compounds of the first line perfusate solution [22,23] and leukocyte depletion [9,22,24] were described. In rats [25] and in rabbits [26] it was reported that PCR improved lung function after ischaemia. PCR was previously studied in rats following 24 hours cold ischaemia, but has not been investigated following warm ischaemia [19].

In this model of an isolated buffer-perfused rabbit lung the effects of different PCR periods on IR-injury following warm hypoxic ischaemia were studied.

## Methods

### Reagents

Sterile Krebs-Henseleit buffer (KHB, Serag-Wiessner, Naila, Germany) was used. The buffer contained (in mM) 125 NaCl, 4.3 KCl, 1.1 KH_2_PO_4_, 2.4 CaCl_2_, 1.3 MgCl_2_, and 13.3 glucose; NaHCO_3 _was adjusted for a constant pH in the range of 7.35–7.45. Gas mixtures for ventilation containing 95%O_2_/5%CO_2_, 5%CO_2_/95%N_2 _and 5%CO_2_/6%O_2_/89%N_2 _were obtained from Linde Gas AG (Düsseldorf, Germany).

### Animal care

The study was performed in accordance to the German laws for animal health and protection declaration and was approved by the local authorities, Government of Rheinlandpfalz (Reg.-Nr.: 177-07/991-3). We used male White New Zealand rabbits of 2500–4000 g body weight (conventional, normally fed ad libitum; Charles River, Kisslegg, Germany). All animals received human care in compliance with the European Convention on Animal Care.

### Lung preparation and experimental setup

Rabbits were anaesthetized with intramuscular xylazine (5–10 mg/kg) and pentobarbital (30 mg/kg) via an ear vein. Tracheal intubation was performed through a tracheostomy and mechanical ventilation was started (Ventilator, Hugo Sachs Electronics Harvard Apparatus, March, D) with room air at a tidal volume of 10 ml/kg and a rate of 50 breaths per minute. The positive endexpiratory pressure (PEEP) was adjusted to 2 cm H_2_O.

Following a median sternotomy and thymectomy the pericardium was incised and the pulmonary artery (PA) and the aorta were dissected free and encircled.

Two purse string sutures were placed into the free walls of the right and left ventricle and heparin was administered intravenously (500 U/kg). The PA and the left atrium (LA) were cannulated via the right and left ventricles. The aorta was ligated and the PA-cannula sutured tightly avoiding perfusate backflow. Perfusion with sterile KHB was started. Room air ventilation was changed to a gas mixture of 95% oxygen and 5% carbon dioxide to preserve the physiological acid-base balance. The lungs were excised while being ventilated and perfused with buffer solution. After rinsing the lungs with 400 ml buffer solution for washout of blood, the perfusion circuit was closed for recirculation (total volume of 500 ml). The flow was adjusted to 100 ml/min without exceeding a PA pressure of 5 mmHg. The left artrial pressure was set at 2 mmHg.

**Figure 1:**
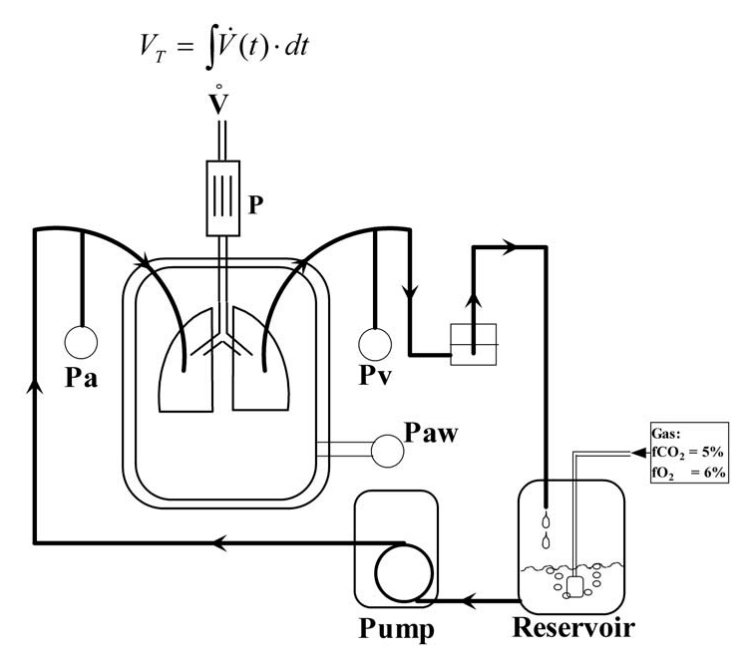
**Set-up**. This figure depicts the experimental model of the isolated buffer-perfused lung. The lungs are mounted in a water-heated glass-chamber ("artificial thorax") that allows for negative pressure ventilation. The negative pressure inside the chamber is measured by a transducer (Paw) and used to calculate the dynamic lung compliance. The tidal volume is calculated by integration of the flow signal of the pneumotachograph (P). The buffer solution is stored in the reservoir and equilibrated with a gas mixture that contained carbon dioxide (5%) and oxygen (6%). The solution is pumped by a roller pump and the flow is pressure-controlled regulated by a software program. The pressure transducers (arterial Pa, venous Pv) are adjusted to the height of the hilum.

**Figure 2:**
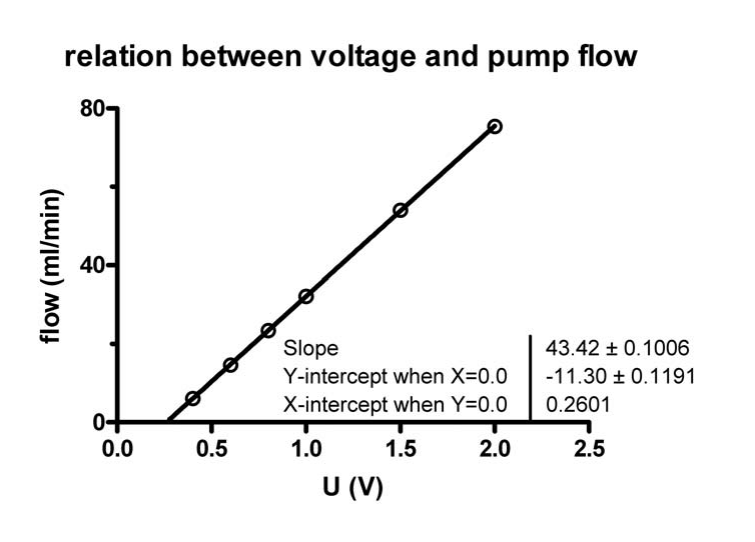
**Flow calibration**. Relation between the voltage (U) and the pump flow (F). Values are means of four measurements. The pump was calibrated for each individual experiment by measuring the volume after 15 minutes of pumping at a voltage of 1 Volt.

Figure 1 shows the heart-lung-block mounted in a temperature-equilibrated artificial thorax allowing negative pressure ventilation (respiratory rate = 50 breaths/min; PEEP = -3 cmH_2_O; inspiratory pressure = -12 cmH_2_O, deep inspiratory pressure = -20 cmH_2_O each minute) with a pressure-constant ventilator (Hugo Sachs Electronics Harvard Apparatus, March, D). Temperature was kept at 38°C. The pressures in the pulmonary artery and the left atrium were registered by small-diameter tubing within the perfusion catheter. A special pressure transducer (Validyne, Hugo Sachs Elektronik – Harvard Apparatus, March, Germany) was used to measure the pressure in the artificial thorax. An assessment program (own software development) regulated the flow of the perfusate allowing for constant pressure or constant flow perfusion. Calibration showed a maximum deviation of ± 3% (Figure 2).

### Assessment

After a stabilisation period all baseline values were assessed. Perfusion solution of the inflow and the outflow was sampled for gas and pH analysis. Mean pulmonary arterial and venous pressures (PAP, PVP), perfusate flow (F_P_), tidal volume (V_T_) and inspiratory and endexpiratory pressures (P_I_, Peep) were measured. Pulmonary vascular resistance (PVR) and compliance of the lung (C_L_) were calculated in real-time. Microvascular pressure (P_C_) was determined by the arterial and venous double occlusion technique [27,28]. Occlusion manoeuvres were performed in end-expiration with electromagnetic tube-clamping devices, synchronised by a D/A-A/D converter connected to a personal computer. This converter allowed parallel data collection at a rate of 100 Hz, further processed by an analysis program (GraphPad Prism version 4.00 for Windows, GraphPad Software, San Diego, CA USA).

Vascular compliance was determined by venous occlusion technique (occlusion time, 1 s). After the early steep increase a constant low increase of pressure was detected. The reciprocal value of this slope of the low pressure increase multiplied with the known perfusion flow allows calculating the vascular compliance.

Lungs included in this study had a homogeneous white appearance without signs of oedema or atelectasis. Further, they had pulmonary artery pressures and lung compliance in the normal range, and were stable during a steady state period of 15 minutes.

### Groups

25 male New Zealand White rabbits (2.5–4 kg) were randomly assigned to experimental groups (n = 5 in each group):

Control group: no ischaemia

All other groups had 2 hours of warm (38°C) and hypoxic ischaemia (PO_2 _= 50–60 mmHg)

Group A: no pressure-controlled reperfusion (PCR), i.e. reperfusion by baseline flow

Group B: 5 minutes of PCR followed by baseline flow

Group C: 15 minutes of PCR followed by baseline flow

Group D: 30 minutes of PCR followed by baseline flow

### Experimental protocol

**Figure 3:**
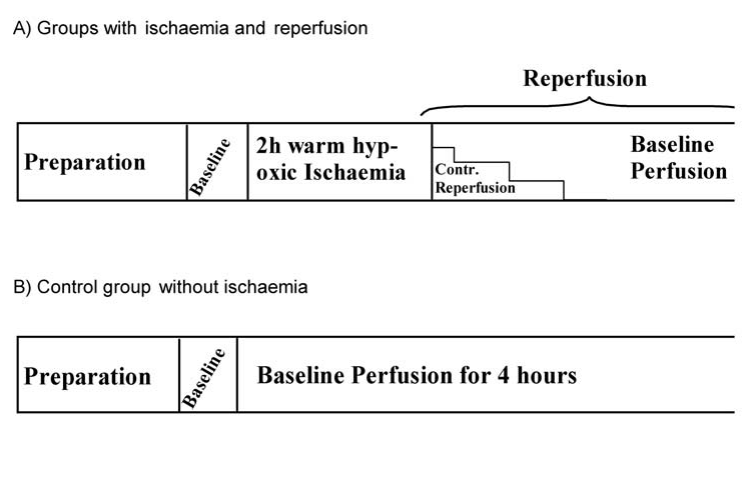
**Protocol**. This figure depicts the experimental protocol for all rabbits. Figure 3A depicts the protocol for pressure-controlled reperfusion. After preparation and at least 15 minutes of stabilization all baseline values were measured. Two hours of warm hypoxic ischaemia followed. Then ventilation and reperfusion started, but with different intervals of pressure-controlled reperfusion from 0 to 30 minutes. Figure 3B depicts the protocol for the control group. After preparation and at least 15 minutes of stabilization all baseline values were measured. Two hours with normal ventilation and perfusion ensued without ischaemia followed by another two hours for measurements.

After the early steady state period (15 minutes) all baseline values were measured (Figure 3). After these measurements the ventilation gas was switched to an anoxic gas mixture (5% CO_2 _in nitrogen) to reduce the alveolar oxygen tension. After 10 minutes of anoxic ventilation perfusate gas values were assessed and ventilation and perfusion were stopped. The outflow tube was clamped and the intravascular pressure adjusted to 5–6 mmHg by slowly pumping of the perfusate. Then also the inflow tube was clamped to maintain a positive intravascular pressure [29]. During 2 hours of warm (38°C) hypoxic ischaemia a negative pressure of -3 cmH_2_O in the artificial thorax was preserved. Just before reperfusion ventilation was started again and the lungs were deeply inflated by lowering the inspiratory pressure down to -20 cmH_2_O. Reperfusion was started after full expansion of the lung.

Group A lungs were immediately reperfused by the baseline flow within 15 seconds. In groups B to D the reperfusion started pressure-controlled limited to a maximum perfusion pressure of 5 mmHg for the period of PCR. Thereafter lungs were perfused using baseline flow. Measurements followed immediately at the beginning (0 minute) and after 5, 15, 30, 60, 90 and 120 minutes of reperfusion. At the end of the experiment lung specimen were frozen in liquid nitrogen and stored at -80°C.

### Wet-to-dry weight ratio

Lung wet-to-dry weight (W/D) ratio was used as a measurement of pulmonary oedema. Samples of lung tissue were weighed and a drying period of 48 h at 80°C ensued. The weight immediately following reperfusion and the weight after drying were used to calculate the lung wet-to-dry weight ratio.

### Myeloperoxidase activity

Quantitative myeloperoxidase activity was determined using the adapted procedures of Okabayashi and associates [30]. Ten to 15 mg of dry lung tissue were homogenised in 1 ml of 0.5% hexadecyltrimethylammonium bromide, 5 mmol/L EDTA, and 50 mmol/L potassium phosphate buffer (pH 6.2) with a tissue grinder (Ultra turrax T18, Ika, Staufen, Germany). The homogenate was centrifuged at 10,000 g for 15 minutes at 4°C. The supernatant was assayed for total soluble protein by the method of Pierce Laboratories. Fifty microliters of supernatant were combined with 0.6 ml Hanks bovine serum albumin, 0.5 ml of 100 mmol/L potassium phosphate buffer (pH 6.2), 0.1 ml 0.05% H_2_O_2_, and 0.1 ml of 1.25 mg/ml o-dianisidine. The optical density was measured at 460 nm (M4QII, Carl Zeiss, Oberkochen, Germany). Colour development from 5 to 20 minutes is linear. Enzyme activity is expressed as the change in optical density units per minute per milligram of tissue protein (δOD/min/mg protein).

### Statistics

All values are expressed as means ± standard deviation. Statistical analysis was performed using the software package GraphPad Prism 4.0 (GraphPad Prism version 4.00 for Windows, GraphPad Software, San Diego, CA, USA). Individual means between groups were compared by 1-way analysis of variance with Bonferroni correction for multiple comparisons. Oxygenation, lung compliance and haemodynamic parameters during the reperfusion period were analysed using 2-way analysis of variance with Bonferroni correction for multiple comparisons. P-values less than 0.05 were considered as significant.

## Results

### Oxygenation capacity

**Figure 4:**
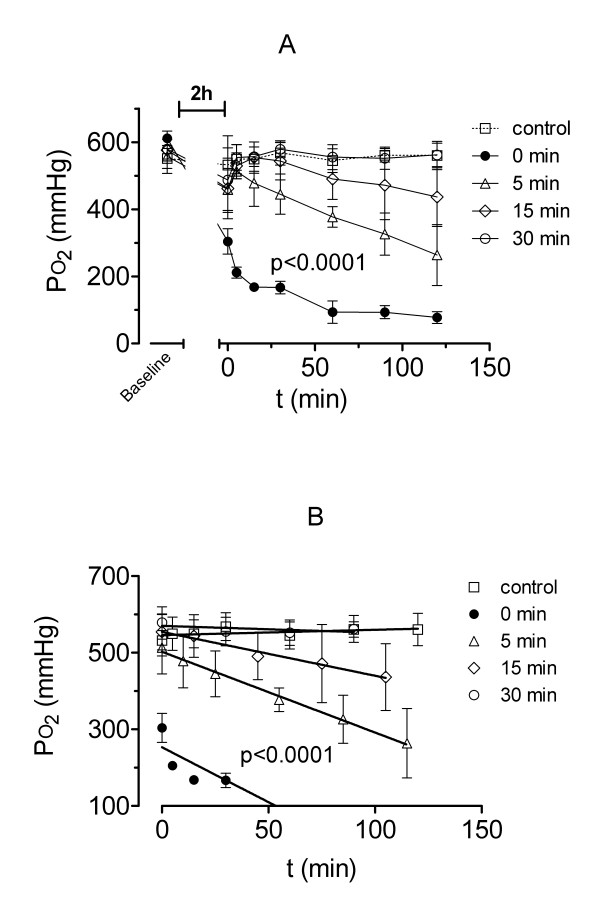
**Oxygen partial pressure**. Oxygen partial pressure (PO_2_, A) and course of PO_2 _with the beginning of reperfusion by baseline flow (B) during 2 hours of isolated buffer-perfused rabbit lungs following 120 min of warm hypoxic ischaemia, except for control animals. Rabbits of group A (●) had no pressure-controlled reperfusion (PCR), whereas rabbits of group B (△) had 5, C (◇) 15 or D (○) 30 minutes of PCR, respectively. Values are presented as means ± standard deviation. Overall differences between groups are presented (see text for details on post-hoc pairwise comparisons).

The difference in oxygen partial pressure (PO_2_) between groups at successive intervals was significant (p < 0.0001), signalling a lasting impairment in oxygenation capacity of most groups due to IR-injury (Figure 4). The overall difference in PO_2 _between groups was also significant (p < 0.0001) as well as the interaction between time and group (p < 0.0001), pointing out the decline in oxygenation capacity which was different between groups because of different grade of IR-injury. Lungs of group A without PCR developed a severe damage and immediate oedema formation (Figure 4A) with a significant decrease in PO_2 _to 77 ± 18 mmHg (p < 0.001). In contrast, PCR resulted in a variable oxygen transfer. There was a significant decrease in PO_2 _for group B (5 min PCR) to 264 ± 91 mmHg (p < 0.001 compared with control group) and in group C (15 min PCR) with statistical significance after 30 to 120 min with 436 ± 87 mmHg compared with 561 ± 42 mmHg of control group (p < 0.001). Only group D (30 min PCR) preserved a high PO_2 _during the whole perfusion period (562 ± 35 mmHg) without a significant difference to the control group. At 120 min groups A to C had also significant lower PO_2 _values compared with group D (p < 0.01).

Figure 4B depicts the shapes of PO_2 _changes over time for all groups starting with the time point of reperfusion by baseline flow. The linear regression functions for group A to C show significant different slopes compared with the control group (p < 0.0001).

### Compliance of the lung

**Figure 5:**
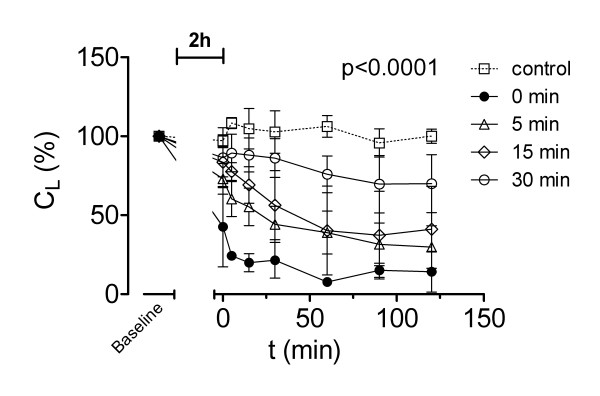
**Lung compliance**. Lung compliance (C_L_) during 2 hours of isolated buffer-perfused rabbit lungs following 120 min of warm hypoxic ischaemia, except for control animals. Rabbits of group A (●) had no pressure-controlled reperfusion (PCR), whereas rabbits of group B (△) had 5, C (◇) 15 or D (○) 30 minutes of PCR, respectively. Values are presented as means ± standard deviation. Overall differences between groups are presented (see text for details on post-hoc pairwise comparisons).

Lung compliance (C_L_) was only stable in the control group over time (Figure 5). All other groups showed a decrease of C_L _after ischaemia. The differences in C_L _within groups at successive time intervals of reperfusion and between groups were significant (p < 0.0001). Without PCR (group A) there was a steep decrease in C_L _down to 15 ± 13% of the baseline values. After ischaemia 30 minutes of PCR (group D) showed the highest C_L_. However, also in this group a significant decrease (76 ± 12%) compared with the control group (106 ± 7%) was found after 60 minutes (p < 0.05). This difference remained stable until the end of the experiment with 70 ± 18% (group D) compared with 100 ± 5% (control group, p < 0.05). Group B and C showed also a significant loss of compliance to 30 ± 13% and 41 ± 27%, respectively (p < 0.001). This was also significantly lower compared with group D (p < 0.01).

### Haemodynamics

#### Mean pulmonary artery pressure

**Figure 6:**
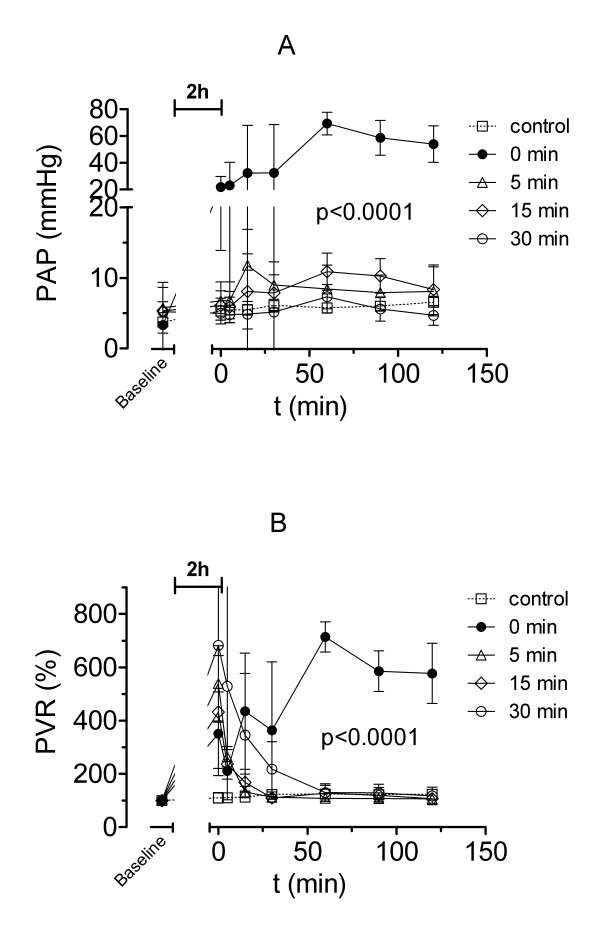
Mean pulmonary arterial pressure and pulmonary vascularresistance Mean pulmonary arterial pressure (PAP, A) and pulmonary vascular resistance (PVR, B) during 2 hours of isolated buffer-perfused rabbit lungs following 120 min of warm hypoxic ischaemia, except for control animals. Rabbits of group A (●) had no pressure-controlled reperfusion (PCR), whereas rabbits of group B (△) had 5, C (◇) 15 or D (○) 30 minutes of PCR, respectively. Values are presented as means ± standard deviation. Overall differences between groups are presented (see text for details on post-hoc pairwise comparisons).

Mean pulmonary artery pressure (PAP) was significantly different between groups (p < 0.0001). The differences within groups at successive time intervals of reperfusion were also significant (p = 0.0227). Figure 6A shows that lack of PCR resulted in a significant increase of PAP to 53 ± 14 mmHg at the end of the experiment (p < 0.001). All other groups with PCR developed also a transient increase in PAP with the highest value of 11.8 ± 5.1 mmHg for group B. However, these differences reached no significance compared to the control group.

#### Pulmonary vascular resistance (PVR)

The differences between groups and within groups at successive time intervals of reperfusion were significant (p < 0.0001). Without PCR (group A) PVR increased progressively (Figure 6B) and reached 755 ± 113% compared with baseline values (p < 0.001). All groups with PCR developed a significant early rise in PVR up to 539 ± 141% (group B), 432 ± 211% (group C) and 682 ± 288% (group D), respectively (p < 0.01). But then in all PCR groups PVR decreased continuously. At the end of the experiment the baseline was reached. Despite a steady decrease of PVR in group D until 15 minutes of PCR a significant elevation with 345 ± 230% of PVR compared with the control group with 113 ± 11% (p < 0.05) was found.

#### Perfusate flow

**Figure 7:**
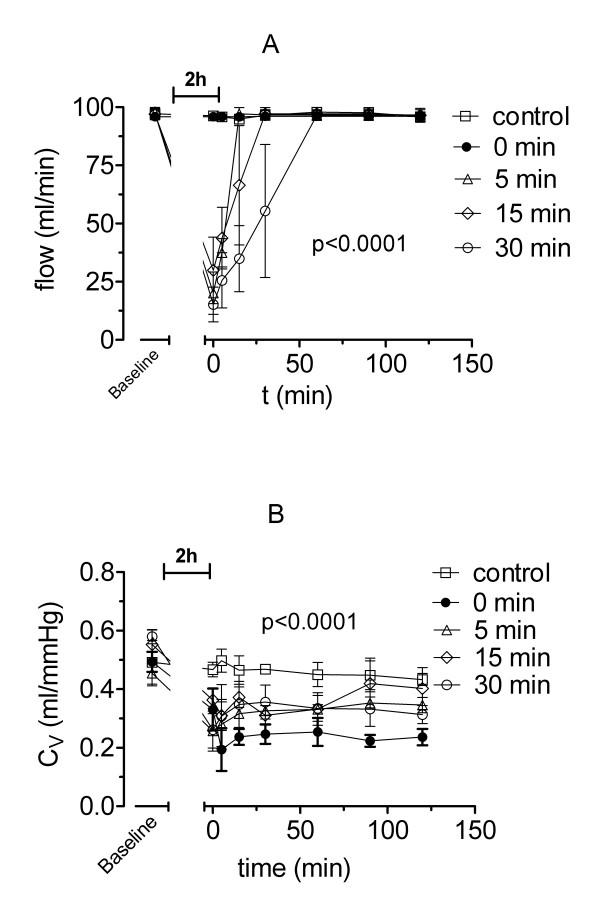
**Perfusate flow and vascular compliance**. Perfusate flow (A) and vascular compliance (C_V_, B) measured by venous occlusion technique during 2 hours of isolated buffer-perfused rabbit lungs following 120 min of warm hypoxic ischaemia, except for control animals. Rabbits of group A (●) had no pressure-controlled reperfusion (PCR), whereas rabbits of group B (△) had 5, C (◇) 15 or D (○) 30 minutes of PCR, respectively. Values are presented as means ± standard deviation. Overall differences between groups are presented (see text for details on post-hoc pairwise comparisons).

The differences between groups and within groups at successive time intervals of reperfusion were significant (p < 0.0001). The perfusate flow (Figure 7A) during PCR was significant reduced compared with baseline perfusion (p < 0.001). However, the values in all PCR groups were highly variable.

#### Vascular compliance

The differences between groups and within groups at successive time intervals of reperfusion were significant (p < 0.0001), reflecting a sharp decrease in vascular compliance (C_V_). After ischaemia and reperfusion there was a significant decrease in C_V _(Figure 7B) approximately down to 50% of the baseline values (p < 0.003). After 15 minutes all PCR groups showed improvement in C_V_. After 90 minutes there was still a lower C_V _compared to baseline for all PCR groups, but the differences did not reach significance.

### Wet-to-dry weight ratio (W/D-ratio)

**Figure 8:**
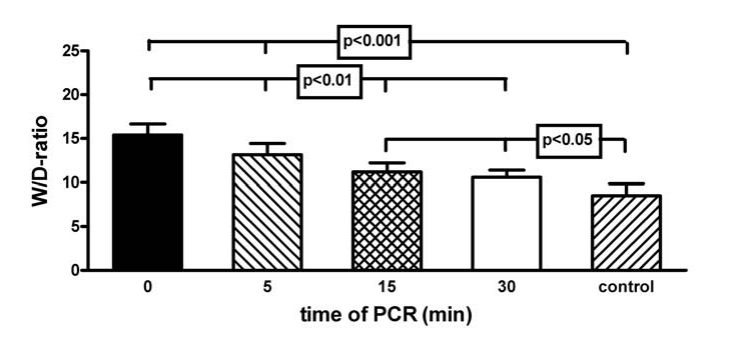
**Wet-to-dry weight ratio**. Wet-to-dry weight (W/D) ratios after 2 hours of reperfusion of isolated buffer-perfused rabbit lungs following 120 min of warm hypoxic ischaemia, except for control animals. Values are presented as means ± standard deviation. Statistically analysis was done by one-way analysis of variance.

Uncontrolled reperfusion resulted in severe oedema formation (Figure 8) indicated by a high W/D-ratio of 15.4 ± 1.2. PCR reduced significantly the oedema formation with the lowest value for group D with 9.8 ± 0.8 (p < 0.001 compared with group A).

### Myeloperoxidase assay

**Figure 9:**
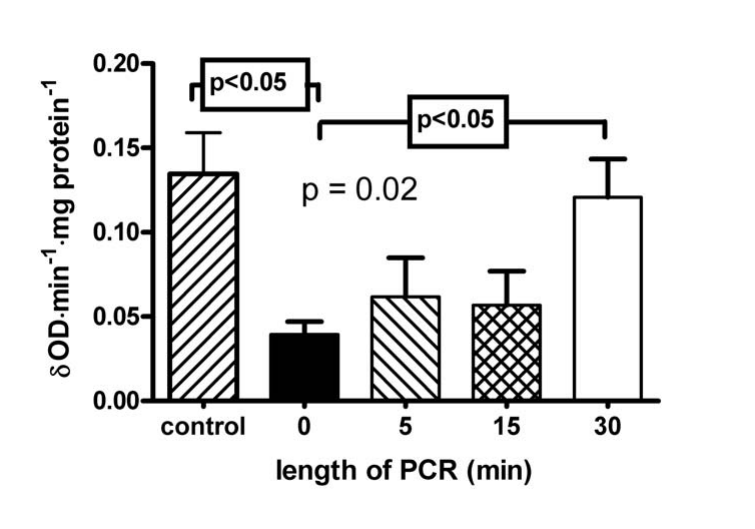
**Myeloperoxidase activity**. Myeloperoxidase activity after 2 hours of reperfusion of isolated buffer-perfused rabbit lungs following 120 min of warm hypoxic ischaemia. Values are presented as means ± standard deviation. Statistically analysis was done by one-way analysis of variance.

Myeloperoxidase activity (Figure 9) of resident lung leukocytes was significantly higher in group D than in group A (0.040 ± 0.008 vs. 0.121 ± 0.055 δOD/min/mg protein, p < 0.05). Also groups B and D showed higher values than group A but these differences did not reach significance. The values of the control group and group D were comparable (0.135 ± 0.060 vs. 0.121 ± 0.055 δOD/min/mg protein).

## Discussion

This study examined the effects of pressure-controlled reperfusion (PCR) on lung IR-injury in a model of warm ischaemia and reperfusion of the isolated buffer-perfused rabbit lung. Uncontrolled reperfusion (immediate restoration of the baseline flow) leads to severe oedema formation, rapid decrease in oxygenation capability (decrease in PO_2_) and lung compliance (C_L_). This was accompanied by a massive increase of pulmonary arterial pressure (PAP) and pulmonary vascular resistance (PVR). These findings are in concordance with other studies which also demonstrated severe lung injury after uncontrolled reperfusion in rabbits [14,26], rats [25] and pigs [31]. All groups with pressure-controlled reperfusion showed less damage in early lung function similarly to other reports [22,25,26,32].

The aim of this study was to investigate the optimal length of PCR to reduce IR-injury. It is clearly shown that 30 minutes of PCR (group D) were superior in attenuating the early IR-injury of the lungs. Only lungs in group D remained a high oxygenation capacity, developed only a slight decrease in C_L _and showed the lowest values of PAP and PVR. But vascular compliance (C_V_) declined in all ischaemic groups compared with baseline suggesting some endothelial dysfunction.

Shorter periods of PCR were less effective in protecting the lungs against IR-injury probably because of severe endothelial dysfunction and lung function progressively worsened.

These data suggest that endothelial permeability in the ischaemic lung was at least increased during the first 15 minutes. Therefore, controlled early reperfusion pressure in our model should exceed 15 minutes. After that time recovery of the endothelial function is satisfactory to prevent the lungs from severe oedema formation.

Our results are in contrast to the data from Bhabra and colleagues [25] who reported in a rat transplantation model that already 10 minutes of PCR prevented the lungs from severe IR-injury. The main differences to our model are (1) the use of a whole animal model, (2) cold storage of the lungs before reperfusion, (3) positive versus negative pressure ventilation, and (4) a different species. So, these reasons may contribute to the difference in the periods of PCR that are necessary for an effective lung protection. Fiser and others showed in rabbits some improvement of lung function after pressure-controlled reperfusion, but this period lasted for only 5 minutes and this was not enough to preserve the oxygenation capability of the lungs [33].

### Ischaemia-reperfusion injury

IR-injury remains a major threat in clinical lung transplantation [2,6,7,11,16,34]. Although we do not know the exact mechanisms of this phenomenon, clinical studies have suggested that patients with pre-existing pulmonary hypertension are at increased risk for reperfusion injury after lung transplantation [31,32,35].

Recently, studies described endothelial cell damage as a trigger that leads to total organ failure, but the cause of this injury is multifactorial [16-18,36]. One important cause is the renewed oxygen supply to the ischaemic organ during early reperfusion provoking formation and activation of various humoural mediators of injury and inflammation [18]. These humoural factors cause an early endothelial dysfunction that leads to marked decline in nitric oxide and prostaglandin release [18].

In addition, an increased hydrostatic gradient across endothelial membranes increases the reperfusion injury in patients with pulmonary hypertension. The accompanying endothelial disruption results in collagen exposure, which may activate leukocytes against the lung tissue and contribute to the severe lung damage [9,37].

### Controlled reperfusion

Controlled organ reperfusion is well known and several experimental studies have been performed to minimize the IR-injury [22,25,30,37-39]. Most have centred on adding substrate to the flush solution, such as oxygen-derived free radical scavengers [8], agents that block neutrophil-endothelial interactions [9,40], endothelial protective agents [33], or haemodilution [39].

Other reports focus on manipulating the onset of reperfusion. Allen et al. described the beneficial effect of regional substrate-enriched blood cardioplegic reperfusion after 4 hours of warm regional ischaemia of the heart [41]. Also in acute limb ischaemia application of controlled reperfusion lessened the reperfusion response [20,21]. Some reports described the effect of controlled reperfusion in lung ischaemia. In these studies the authors have shown that reperfusion with low initial PAP at least partially decreased IR-injury [25,26,32,42,43]. But in these experiments PCR was almost only one part in the concept of controlled reperfusion and it was combined with other methods like leukocyte depletion, adding protective agents, or varying the composition of the perfusate solution. So, the importance of the length of PCR is not clear in the concept of controlled reperfusion.

In our study we focused only on the length of PCR. So, we were able to determine the effects of PCR on ischaemic lungs unaffected by other therapeutic options.

With different periods of PCR lungs showed a gradually improved function with only slight damage in group D. But especially figures 6B and 7A depict that after 30 minutes of PCR there was also a significant decrease in perfusate flow and an increase in PVR suggesting still a functional injury of the vasculature.

The intrapulmonary leukocyte pool and blood leukocytes are considered as a trigger of IR-injury [24,44-46]. Although many investigations have confirmed the role of neutrophils in reperfusion injury [9,24,44,47], other have questioned neutrophil involvement [48]. One report of Fiser et al. could show that in the earliest phase of reperfusion injury the donor (resident) pulmonary leukocyte pool plays an important role [46]. They blocked the pulmonary macrophages with Gadolinium chloride, a rare lanthanide earth salt, which significantly attenuates at least the earliest phase (30 min) of reperfusion injury. In our experiments we were able to focus on the intrapulmonary leukocyte pool because perfusion consisted of KHB-solution adding no other blood cells.

The lung capillaries harbour large pools of immunocompetent cells. Neutrophils represent the largest intracapillary population followed by lymphocytes and monocytes [45]. Interestingly, we could show that after 30 min of PCR myeloperoxidase activity was significantly higher compared with lungs without PCR signalling that much more resident leukocytes were present in these less harmed lungs. So, one might speculate that long-lasting PCR prevents the intrapulmonary leukocytes against activation which attenuates reperfusion injury.

At the moment, the effects of PCR during early reperfusion are not well understood. The reduced perfusion pressure (1) might decrease endothelial disruption, (2) prevent activation of resident intrapulmonary leukocytes (3) stabilize the endothelial function, and (4) improve restoration of normal endothelial and muscular integrity. This might be important in all patients with marked pulmonary hypertension, especially after LTX for primary or secondary pulmonary hypertension [35]. Future experiments should focus on the endothelial muscular integrity of the pulmonary vasculature to further elucidate the mechanisms of PCR.

### Limitations

Like other animal models our model has limitations. First, we used the isolated buffer-perfused rabbit lung and not a whole animal model. But the advantages are (1) focusing on the lung, (2) constancy of circulatory and ventilatory parameters, and (3) avoidance of immunological phenomena that could not be ruled out in whole animal transplantation models.

Further, we are using the model of warm hypoxic ischaemia in contrast to the standard of cold ischaemia in LTX. However, a recent report from Warnecke and co-workers inferred no qualitative difference between these two types of ischaemia [49]. So both models are utilizable in experiments focusing on IR-injury.

### Clinical implications

Our results strongly recommend to applicate PCR after prolonged lung grafts ischaemia. In our study at least 15 to 30 minutes of PCR are required to guarantee a satisfactory organ function. In the clinical setting this is obviously time-consuming, but adding other modalities like inhalative application of prostaglandins, or application of poly-ADP ribose synthetase inhibitors to the concept of pressure controlled reperfusion may further ameliorate IR-injury of the lungs. Combining several factors of controlled reperfusion should lessen the necessary length of PCR. Several measures of controlled reperfusion can hopefully reduce lR-injury of the lungs and may decrease the risk of LTX. The pool of possible organ donors may be extended.

## Conclusion

Our study demonstrates the positive effects of PCR on pulmonary IR-injury. The length of PCR is critical for early lung function and a period of 30 minutes is effective to keep acceptable oxygenation ability. But recovery of the endothelial muscular function of the pulmonary vasculature was not complete. Further experiments are warranted to discover the synergistic effects of PCR with other protective strategies and to clarify the positive effects on the pulmonary vasculature.

## Competing interests

The author(s) declare that they have no competing interests.

## Authors' contributions

SG had the idea of the study, did the surgery on the animals, performed data analysis, statistics, graphics, and wrote the paper. DP amended the study design, data interpretation, and helped to draft the manuscript. TK did the surgery on the animals and investigations. EM helped with the surgical techniques, co-wrote the manuscript and added important comments to the paper. All authors read and approved the final manuscript.

## References

[B1] Macdonald P, Mundy J, Rogers P, Harrison G, Branch J, Glanville A, Keogh A, Spratt P (1995). Successful treatment of life-threatening acute reperfusion injury after lung transplantation with inhaled nitric oxide. J Thorac Cardiovasc Surg.

[B2] Chatila WM, Furukawa S, Gaughan JP, Criner GJ (2003). Respiratory failure after lung transplantation. Chest.

[B3] Christie JD, Van Raemdonck D, de Perrot M, Barr M, Keshavjee S, Arcasoy S, Orens J (2005). Report of the ISHLT Working Group on Primary Lung Graft Dysfunction part I: introduction and methods. J Heart Lung Transplant.

[B4] Christie JD, Carby M, Bag R, Corris P, Hertz M, Weill D (2005). Report of the ISHLT Working Group on Primary Lung Graft Dysfunction part II: definition. A consensus statement of the International Society for Heart and Lung Transplantation. J Heart Lung Transplant.

[B5] Christie JD, Kotloff RM, Pochettino A, Arcasoy SM, Rosengard BR, Landis JR, Kimmel SE (2003). Clinical risk factors for primary graft failure following lung transplantation. Chest.

[B6] de Perrot M, Liu M, Waddell TK, Keshavjee S (2003). Ischemia-reperfusion-induced lung injury. Am J Respir Crit Care Med.

[B7] Schmid RA, Yamashita M, Ando K, Tanaka Y, Cooper JD, Patterson GA (1996). Lidocaine reduces reperfusion injury and neutrophil migration in canine lung allografts. Ann Thorac Surg.

[B8] Sasaki S, Alessandrini F, Lodi R, McCully J, LoCicero J (1996). Improvement of pulmonary graft after storage for twenty-four hours by in vivo administration of lazaroid U74389G: functional and morphologic analysis. J Heart Lung Transplant.

[B9] Uthoff K, Zehr KJ, Lee PC, Low RA, Baumgartner WA, Cameron DE, Stuart RS (1995). Neutrophil modulation results in improved pulmonary function after 12 and 24 hours of preservation. Ann Thorac Surg.

[B10] Waddell TK, Gorczynski RM, DeCampos KN, Patterson GA, Slutsky AS (1996). Major histocompatibility complex expression and lung ischemia-reperfusion in rats. Ann Thorac Surg.

[B11] Fiser SM, Tribble CG, Long SM, Kaza AK, Kern JA, Jones DR, Robbins MK, Kron IL (2002). Ischemia-reperfusion injury after lung transplantation increases risk of late bronchiolitis obliterans syndrome. Ann Thorac Surg.

[B12] Van Raemdonck DE, Jannis NC, De Leyn PR, Flameng WJ, Lerut TE (1998). Alveolar expansion itself but not continuous oxygen supply enhances postmortem preservation of pulmonary grafts. Eur J Cardiothorac Surg.

[B13] Gasparri RI, Jannis NC, Flameng WJ, Lerut TE, Van Raemdonck DE (1999). Ischemic preconditioning enhances donor lung preservation in the rabbit. Eur J Cardiothorac Surg.

[B14] Binns OA, DeLima NF, Buchanan SA, Cope JT, King RC, Marek CA, Shockey KS, Tribble CG, Kron IL (1996). Both blood and crystalloid-based extracellular solutions are superior to intracellular solutions for lung preservation. J Thorac Cardiovasc Surg.

[B15] Hidalgo MA, Shah KA, Fuller BJ, Green CJ (1996). Cold ischemia-induced damage to vascular endothelium results in permeability alterations in transplanted lungs. J Thorac Cardiovasc Surg.

[B16] Novick RJ, Gehman KE, Ali IS, Lee J (1996). Lung preservation: the importance of endothelial and alveolar type II cell integrity. Ann Thorac Surg.

[B17] Boyle EM, Pohlman TH, Johnson MC, Verrier ED (1997). Endothelial cell injury in cardiovascular surgery: the systemic inflammatory response. Ann Thorac Surg.

[B18] Lefer AM, Lefer DJ (1993). Pharmacology of the endothelium in ischemia-reperfusion and circulatory shock. Annu Rev Pharmacol Toxicol.

[B19] Bhabra MS, Hopkinson DN, Shaw TE, Hooper TL (1996). Critical importance of the first 10 minutes of lung graft reperfusion after hypothermic storage. Ann Thorac Surg.

[B20] Allen BS, Hartz RS, Buckberg GD, Schuler JJ (1998). Prevention of ischemic damage using controlled limb reperfusion. J Card Surg.

[B21] Beyersdorf F, Sarai K, Mitrev Z, Eckel L, Ihnken K, Satter P (1993). Studies of reperfusion injury in skeletal muscle: controlled limb reperfusion to reduce post-ischaemic syndrome. Cardiovasc Surg.

[B22] Halldorsson AO, Kronon M, Allen BS, Rahman S, Wang T, Layland M, Sidle D (1998). Controlled reperfusion prevents pulmonary injury after 24 hours of lung preservation. Ann Thorac Surg.

[B23] Serrick CJ, Jamjoum A, Reis A, Giaid A, Shennib H (1996). Amelioration of pulmonary allograft injury by administering a second rinse solution. J Thorac Cardiovasc Surg.

[B24] Kurusz M, Roach JD, Vertrees RA, Girouard MK, Lick SD (2002). Leukocyte filtration in lung transplantation. Perfusion.

[B25] Bhabra MS, Hopkinson DN, Shaw TE, Onwu N, Hooper TL (1998). Controlled reperfusion protects lung grafts during a transient early increase in permeability. Ann Thorac Surg.

[B26] Fiser SM, Kron IL, Long SM, Kaza AK, Kern JA, Cassada DC, Laubach VE, Tribble CG (2002). Controlled perfusion decreases reperfusion injury after high-flow reperfusion. J Heart Lung Transplant.

[B27] Audi SH, Dawson CA, Rickaby DA, Linehan JH (1991). Localization of the sites of pulmonary vasomotion by use of arterial and venous occlusion. J Appl Physiol.

[B28] Hakim TS (1988). Identification of constriction in large versus small vessels using the arterial-venous and the double-occlusion techniques in isolated canine lungs. Respiration.

[B29] Schutte H, Hermle G, Seeger W, Grimminger F (1998). Vascular distension and continued ventilation are protective in lung ischemia/reperfusion. Am J Respir Crit Care Med.

[B30] Okabayashi K, Aoe M, DeMeester SR, Cooper JD, Patterson GA (1994). Pentoxifylline reduces lung allograft reperfusion injury. Ann Thorac Surg.

[B31] Halldorsson A, Kronon M, Allen BS, Bolling KS, Wang T, Rahman S, Feinberg H (1998). Controlled reperfusion after lung ischemia: implications for improved function after lung transplantation. J Thorac Cardiovasc Surg.

[B32] Halldorsson AO, Kronon MT, Allen BS, Rahman S, Wang T (2000). Lowering reperfusion pressure reduces the injury after pulmonary ischemia. Ann Thorac Surg.

[B33] Fiser SM, Cope JT, Kron IL, Kaza AK, Long SM, Kern JA, Tribble CG, Lowson SM (2001). Aerosolized prostacyclin (epoprostenol) as an alternative to inhaled nitric oxide for patients with reperfusion injury after lung transplantation. J Thorac Cardiovasc Surg.

[B34] Christie JD, Kotloff RM, Ahya VN, Tino G, Pochettino A, Gaughan C, DeMissie E, Kimmel SE (2005). The effect of primary graft dysfunction on survival after lung transplantation. Am J Respir Crit Care Med.

[B35] Bando K, Keenan RJ, Paradis IL, Konishi H, Komatsu K, Hardesty RL, Griffith BP (1994). Impact of pulmonary hypertension on outcome after single-lung transplantation. Ann Thorac Surg.

[B36] Verrier ED, Boyle EM (1996). Endothelial cell injury in cardiovascular surgery. Ann Thorac Surg.

[B37] Breda MA, Hall TS, Stuart RS, Baumgartner WA, Borkon AM, Brawn JD, Hutchins GM, Reitz BA (1985). Twenty-four hour lung preservation by hypothermia and leukocyte depletion. J Heart Transplant.

[B38] Eppinger MJ, Ward PA, Jones ML, Bolling SF, Deeb GM (1995). Disparate effects of nitric oxide on lung ischemia-reperfusion injury. Ann Thorac Surg.

[B39] Puskas JD, Oka T, Mayer E, Wisser W, Downey GP, Slutsky AS, Patterson GA (1994). Hemodilution reduces early reperfusion injury in an ex vivo rabbit lung preservation model. Ann Thorac Surg.

[B40] Clark SC, Sudarshan C, Khanna R, Roughan J, Flecknell PA, Dark JH (1998). Controlled reperfusion and pentoxifylline modulate reperfusion injury after single lung transplantation. J Thorac Cardiovasc Surg.

[B41] Allen BS, Okamoto F, Buckberg GD, Bugyi H, Young H, Leaf J, Beyersdorf F, Sjostrand F, Maloney JV (1986). Immediate functional recovery after six hours of regional ischemia by careful control of conditions of reperfusion and composition of reperfusate. J Thorac Cardiovasc Surg.

[B42] Lick SD, Brown PS, Kurusz M, Vertrees RA, McQuitty CK, Johnston WE (2000). Technique of controlled reperfusion of the transplanted lung in humans. Ann Thorac Surg.

[B43] Schnickel GT, Ross DJ, Beygui R, Shefizadeh A, Laks H, Saggar R, Lynch JP, Ardehali A (2006). Modified reperfusion in clinical lung transplantation: the results of 100 consecutive cases. J Thorac Cardiovasc Surg.

[B44] Eppinger MJ, Jones ML, Deeb GM, Bolling SF, Ward PA (1995). Pattern of injury and the role of neutrophils in reperfusion injury of rat lung. J Surg Res.

[B45] Ermert L, Duncker HR, Rosseau S, Schutte H, Seeger W (1994). Morphometric analysis of pulmonary intracapillary leukocyte pools in ex vivo-perfused rabbit lungs. Am J Physiol.

[B46] Fiser SM, Tribble CG, Long SM, Kaza AK, Kern JA, Kron IL (2001). Pulmonary macrophages are involved in reperfusion injury after lung transplantation. Ann Thorac Surg.

[B47] Fiser SM, Tribble CG, Long SM, Kaza AK, Cope JT, Laubach VE, Kern JA, Kron IL (2001). Lung transplant reperfusion injury involves pulmonary macrophages and circulating leukocytes in a biphasic response. J Thorac Cardiovasc Surg.

[B48] Deeb GM, Grum CM, Lynch MJ, Guynn TP, Gallagher KP, Ljungman AG, Bolling SF, Morganroth ML (1990). Neutrophils are not necessary for induction of ischemia-reperfusion lung injury. J Appl Physiol.

[B49] Warnecke G, Sommer SP, Gohrbandt B, Fischer S, Hohlfeld JM, Niedermeyer J, Haverich A, Struber M (2004). Warm or cold ischemia in animal models of lung ischemia-reperfusion injury: is there a difference?. Thorac Cardiovasc Surg.

